# A mild, effective, and green micro-dissolution treatment for wool fiber using choline-ascorbic acid NADES

**DOI:** 10.1371/journal.pone.0360539

**Published:** 2026-10-16

**Authors:** Zhe Jiang

**Affiliations:** Public Security School, Liaoning Police College, Dalian City, China; Monash University Malaysia, MALAYSIA

## Abstract

Herein, we proposed a mild and effective approach for the surface modification of wool fiber using a natural deep eutectic solvent (NADES) composed of choline chloride and ascorbic acid (Vitamin C), avoiding the unsustainability of oxidation methods and severe damage of enzyme methods. The results showed that the NADES could remove keratin-associated proteins partly due to the breakage of disulfide bonds with the retention of the protein’s main macromolecular skeleton and secondary structure, and the exposure degree increase of hydrophilic groups. Furthermore, the hydrophily, dye performance, and anti-felting properties were improved and met industrial standards. The developed method had apparent advances in treatment time, energy consumption, and chemical usage compared to present methods. This work contributes to the further investigation of NADES on the protein modification.

## 1. Introduction

Micro-dissolution treatment is a facile and highly effective process for adding the reaction sites and improving the related performance of the material, such as hydrophilic or hydrophobicity, via a mild method with minimum structure loss [1–3]. At present, green and renewable protein-based material is regarded as a positive alternative to petroleum-based material, but the complicated three-dimensional structure, stable crosslink bonds, and noble groups are against further application [4]. For example, the outer scale layer of keratin composite fiber, containing a large amount of keratin-associated proteins, restricts its further application in the material field due to the rich disulfide bonds crosslinking network, although it has the more excellent performance than man-made fiber, such as skin-friendliness, good elasticity and antibacterial property [5,6].

The chlorination method is the widely used method for the cleavage of disulfide bonds using chloride reagents such as dichloroisocyanuric acid (DCCA) and sodium hypochlorite [7]. However, the method could result serve Absorbable Organic Halogen (AOX) pollution in the wastewater [8]. With the development of biotechnology, eco-friendly enzyme, including protease, lipase, and cutinase, is gradually applied for the wool modification [9–11]. However, due to the complicated component, the treatment effect of the enzyme method is not satisfactory. Furthermore, the permeation of small-size enzyme molecular to the fiber’s inner part could result in severe fiber strength loss [12,13]. Ionic liquid (IL) was widely used for protein modification as a green solvent, but the recent investigation indicated that the aromatic ring (such as imidazole) from IL could not be degraded in the natural condition and had a potential risk to the environment [14]. Therefore, it is significant and vital to develop a low-damage and eco-friendly approach for the protein subtractive modification.

As proposed by Abbot et al., deep eutectic solvent (DES) has been regarded as a novel alternative to ionic liquid due to the similar properties between them and the more facile preparation method [15]. As a kind of DES, natural deep eutectic solvent (NADES) has attracted more attention in the field of green chemistry because it is composed of natural products, such as choline, organic acid, and polyol [16]. Previous studies indicated that choline chloride urea DES had a poor effect on protein modification due to the loss of ability to break the disulfide bonds, and the excessive nitrogen application could bring potential environmental risk [17–20].

As a natural water-soluble vitamin, ascorbic acid, also called Vitamin C (VC), is abundant in the tissues of fruits, vegetables, and animals [21]. Ascorbic acid and dehydroascorbic acid (DHAA, reversible oxidized form) form a pair of redox systems, which are used as a reducing agent in animals to remove active oxygen in the body for reducing the damage of active oxygen in cells [22]. Furthermore, DHAA could be easily oxidized in the body, adding an oxygen source and converting into 2, 3-diketogulonic acid (DKG) [23]. At present, ascorbic acid has been widely used in the field of food and cosmetics as an antioxidant [24,25]. However, the application for protein subtractive modification has not been studied.

In this work, the NADES was prepared using choline chloride and ascorbic acid, and applied for the surface modification of wool fiber with minimal damage to its internal structure, aiming at improving the surface-related properties. The morphology of the fiber surface was observed using a scanning electron microscope (SEM) and atomic force microscope (AFM). The structure, including chemical bonds and secondary structure, was characterized by Fourier transform infrared spectroscopy (FTIR), nuclear magnetic resonance (NMR), and X-ray photoelectron spectroscopy (XPS). Furthermore, the surface-related properties, including wettability, dye ability, and felting shrinkage, were examined. Finally, the method advance was also investigated by comparison with other methods. This work provides a facile method to modify natural protein materials such as wool and feather, which contributes to further applications of natural reagents in protein performance improvement.

## 2. Materials and methods

### 2.1 Materials

Wool fiber (~24 μm in diameter) was purchased from Ruyi Company (Shandong, China). Ascorbic acid and choline chloride were analytically pure and purchased from Aladdin Company (Shanghai, China).

### 2.2 Methods

#### 2.2.1 Preparation of NADES.

NADES was prepared as follows: 1:1 mixture of choline chloride and ascorbic acid (mol/mol) was dried and mixed at 80 ℃, until the liquid became homogeneous [26].The water content of the NADES was determined to be < 0.5 wt.% from Karl Fischer analysis. The tested viscosity (596.1 cP) at 80 ℃ of the NADES provided the solvent liquidity.

#### 2.2.2 Treatment process.

5 g of wool sample was treated by NADES at a solid-liquid weight ratio of 1:5 for different times (3 and 5 min) at 80 ℃. Then, the sample was obtained by water washing and drying at room temperature.

#### 2.2.3 Surface morphology observation.

The fiber surface morphologies of wool samples were observed by using a SU1510 scanning electron microscope (Hitachi, Japan). The samples were sputter-coated with gold before the test. The test conditions were 15 kV of acceleration voltage and 50 pA of current. A CSPM 5500 atomic force microscopy (Benyuan, China) was used for the three-dimensional surface morphologies observation. Samples were fixed on magnetic sample stubs, and the scanning was carried out at a size of 5000 × 5000 nm and a scanning frequency of 2.0 Hz in tapping mode with a silicon cantilever.

#### 2.2.4 FTIR analysis.

The FTIR spectra were determined on a Nicolet iS10 spectrometer (Thermo Scientific, USA). The test range was from 500 to 4000 cm^-1^ with the 4 cm^-1^ resolution and 32 scans.

#### 2.2.5 ^13^C solid-state NMR spectroscopy.

The NMR spectra of keratin samples were recorded at 400 MHz on an Avance-400 spectrometer (Bruker, Germany). The NMR spectra were acquired using a 4 mm rotor with a Kel-f cap at a spinning rate of 10 KHz. The contact time in CP MAS (cross polarization magic angle spinning) experiments was 2.4 ms with a recycle delay of 1 s and cw decoupling. The number of scans was ∼90 000–100 000.

#### 2.2.6 Raman spectroscopy.

Raman spectroscopy was collected using a 785 nm laser, which was excited by a 50 mW argon ion laser (Renishaw, UK). The laser beam spot diameter on the sample was focused to 1 μm using a 100 × objective, and the spectral scanning area was from 400 to 1800 cm^-1^ region with a total acquisition time of 1000 s.

#### 2.2.7 X-ray photoelectron spectroscopy (XPS).

XPS spectra of keratin samples were carried out using RBD upgraded PHI-5000C ESCA system (Perkin Elmer, USA). General spectra in range of 0–1100 eV over a sample area of 300 × 700 µm and narrow spectra at high resolution for all elements were recorded.

#### 2.2.8 Wetting properties test.

The wetting properties test, including water contact angle and wetting time, were conducted using a contact angle tester. The contact angle was the value of the droplet contacting the sample. The wetting time was also recorded. Each sample was tested 5 times and took an average value.

#### 2.2.9 Dyeing performance.

Dyeing prescription: 2% o.w.f. (on weight the fabric) acid red B (C.I. (color index)249), pH = 4.5, bath ratio 1:50. Wool was soaked in the dye bath and dyed at 80 ℃ for 60 min. Then the dyed sample was washed and dried.

The dye absorption content was ascertained by the color strength (K/S). K/S was measured using a Color-i5 computer colourimeter (X-rite, USA), where K is the absorption coefficient, S is the scattering coefficient of the substrate. Each fabric was folded to four layers and measured at ten different locations, and the average K/S value was calculated.

Color fastnesses were tested according to the AATCC Test Methods 61−2013 (Color Fastness to Water Washing, https://members.aatcc.org/store/tm61/495/), 120−2019 (Color Change to Flat Abrasion, https://members.aatcc.org/store/tm120/530/) and 16−2020 (Colorfastness to Light, https://members.aatcc.org/store/tm16-3/959/), respectively.

#### 2.2.10 Felting ball test.

Using the Aachener 3-D shaking machine, the tendency of wool tops to felt was evaluated according to the IWTO-20–69 standard test method [27]. A 1 g wool sample (pre-conditioned at 20°C and relative humidity 65%) was added into a 100 mL screw-top cylindrical container with 50 mL phosphate buffer pH 7. The container, with its contents, was shaken (150 rpm) in the felting machine for 1 h. Using a digital vernier caliper, the diameter of the obtained dried felt ball was determined in nine different places, and the average value was calculated. The smaller the felt ball diameter is, the higher will be the felt ball density, and greater the felting propensity.

## 3. Results and discussion

### 3.1 Surface topography

The SEM pictures were shown in Fig 1. It can be seen from the picture of raw fiber that it has a dense scale layer, and the outline of the scale layer is evident at the initial time. After the treatment for 3 min, the scale layer was broken partly. When the time increased to 5 min, the scale layer was further broken, and the outline was not apparent. However, the cortex layer of wool fiber is intact. The SEM results indicated that the NADES can modify the wool surface but have little effect on the cortex layer of wool. It also shows that surface modification could improve wool surface performance.To further observe the changes in fiber surface morphology before and after the modification, the AFM photos were taken and listed in Fig 2. For raw fiber, there are apparent protrusions located on the fiber surface. With the treatment time increase, the fiber became relatively smooth.

**Fig 1:**
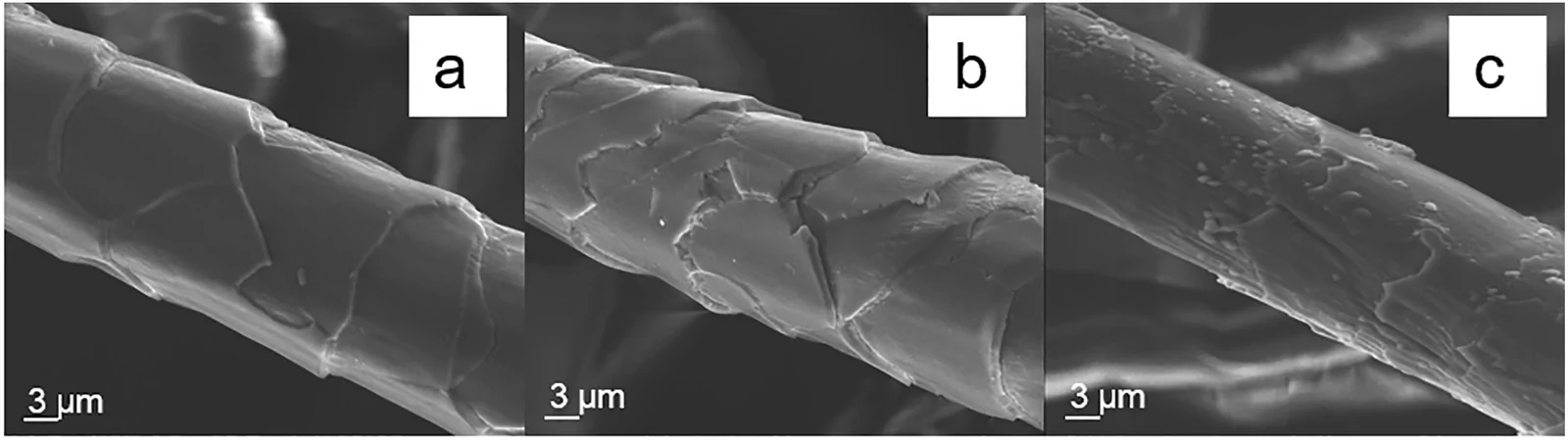
SEM images of raw (a) and modified wool for (b) 3 and (c) 5 min. (Image by Zhe Jiang, 2026, licensed under CC BY 4.0.).

**Fig 2:**
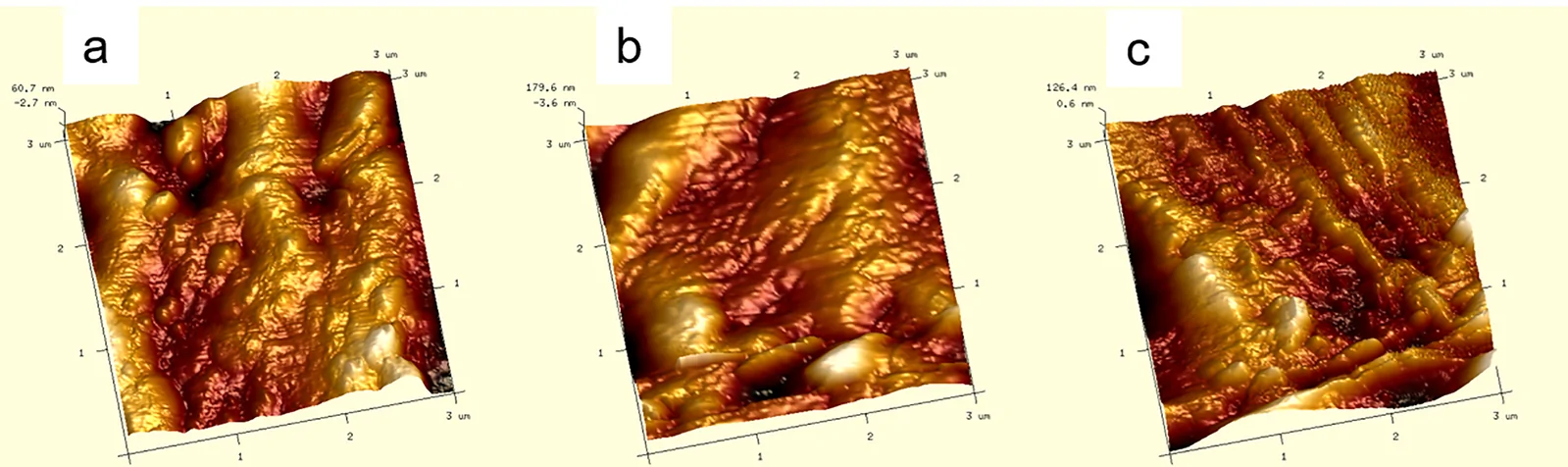
AFM images of raw (a) and treated wool for (b) 3 and (c) 5 min. (Image by Zhe Jiang, 2026, licensed under CC BY 4.0.).

### 3.2 Chemical bonds analysis

The FTIR spectra were collected and shown in Fig 3 for evaluating the effect on the chemical bonds. The peptide bonds are the main chemical bond of wool due to it being a natural protein fiber, which was classified into four components, which were called Amide A (3270 cm^−1^), Amide I (1652–1640 cm^−1^), Amide II (1546–1535 cm^−1^), and Amide III (1268–1255 cm^−1^) [28]. These bands were observed in the FTIR spectra of raw and treated samples, which indicated that the protein backbone was retained and not broken with the treatment time increase.

**Table 1:** Secondary structure of raw and treated sample for 5 min.

Samples		Content/%
Raw wool	α-helix	52.7
	Random coil+β-sheet	47.3
Treated wool	α-helix	51.8
	Random coil+β-sheet	48.2

**Fig 3:**
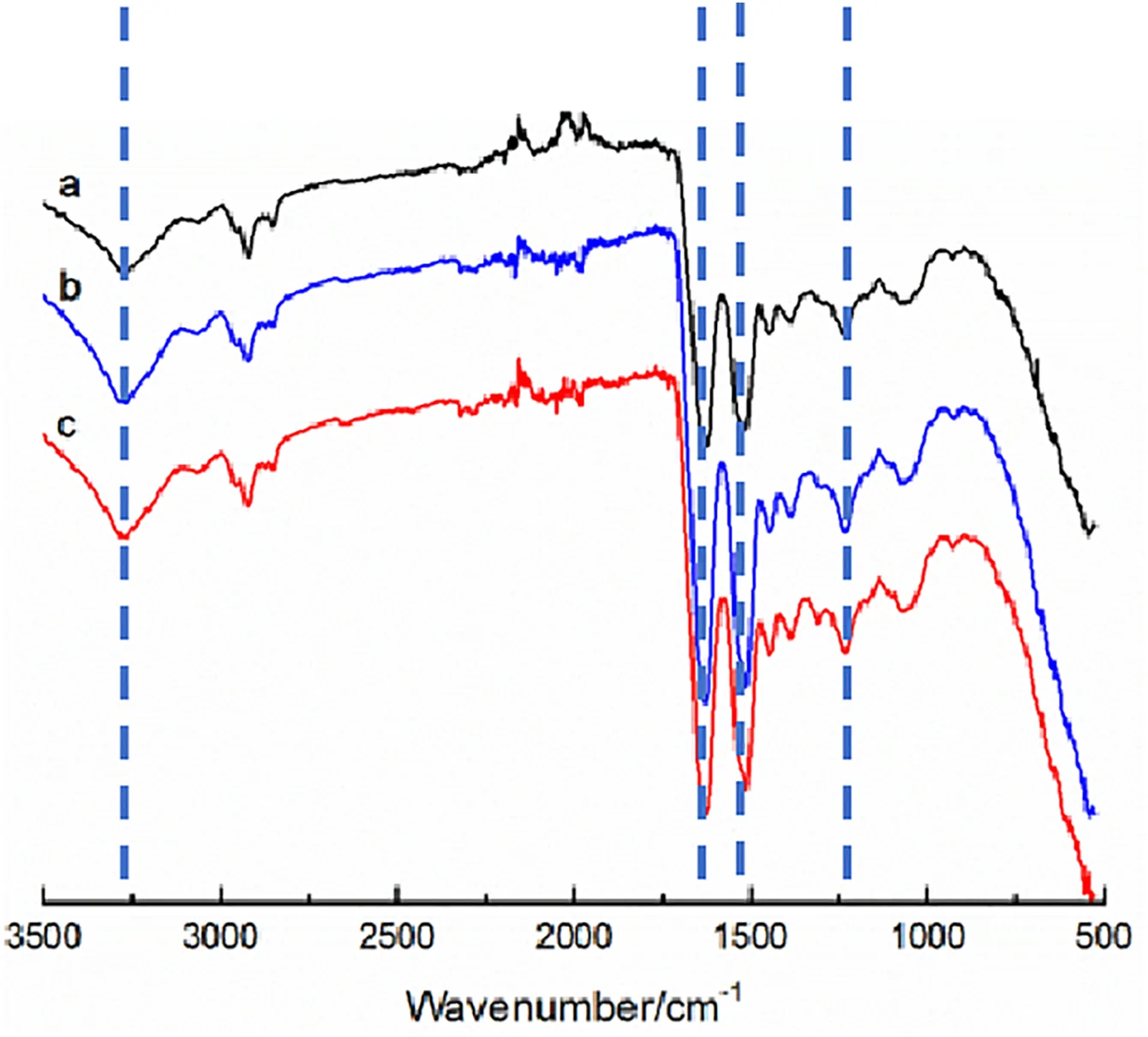
FTIR of raw (a) and treated wool for (b) 3 min and (c) 5 min.

It could be predicted that the secondary structure component could have no obvious change from the FTIR results [29]. Furthermore, the ^13^C NMR spectra of the control and treated samples by 5 min was collected for verifying the result, which were shown in Fig 4. The peak observed at δ = 175 ppm was related to the α-helix structure, and the other peak at δ = 172 ppm can be ascribed to the β-sheet structure [30, 31]. Compared with the 52.7% fraction of the α-helix structure in the raw wool sample, the fraction of the wool sample treated for 5 min solution decreased to 51.9% (Table 1). The result indicated that the treatment process was controlled on the fiber surface and did not break the secondary structure.

**Table 2:** Surface element content of raw wool and treated wool for 5 min.

Samples	Content%			Area (%)					
	C1s	N1s	O1s	S2p	-C-C-, -C-H-	-C-O-, -C-N-	-C = O-	-S-S-	-SO_x_
Raw wool	75.3	7.5	15.1	2.3	65.8	24.1	10.1	83.3	16.7
Treated wool	72.7	9.1	16.8	1.4	61.7	26.5	11.8	74.5	25.5

**Fig 4:**
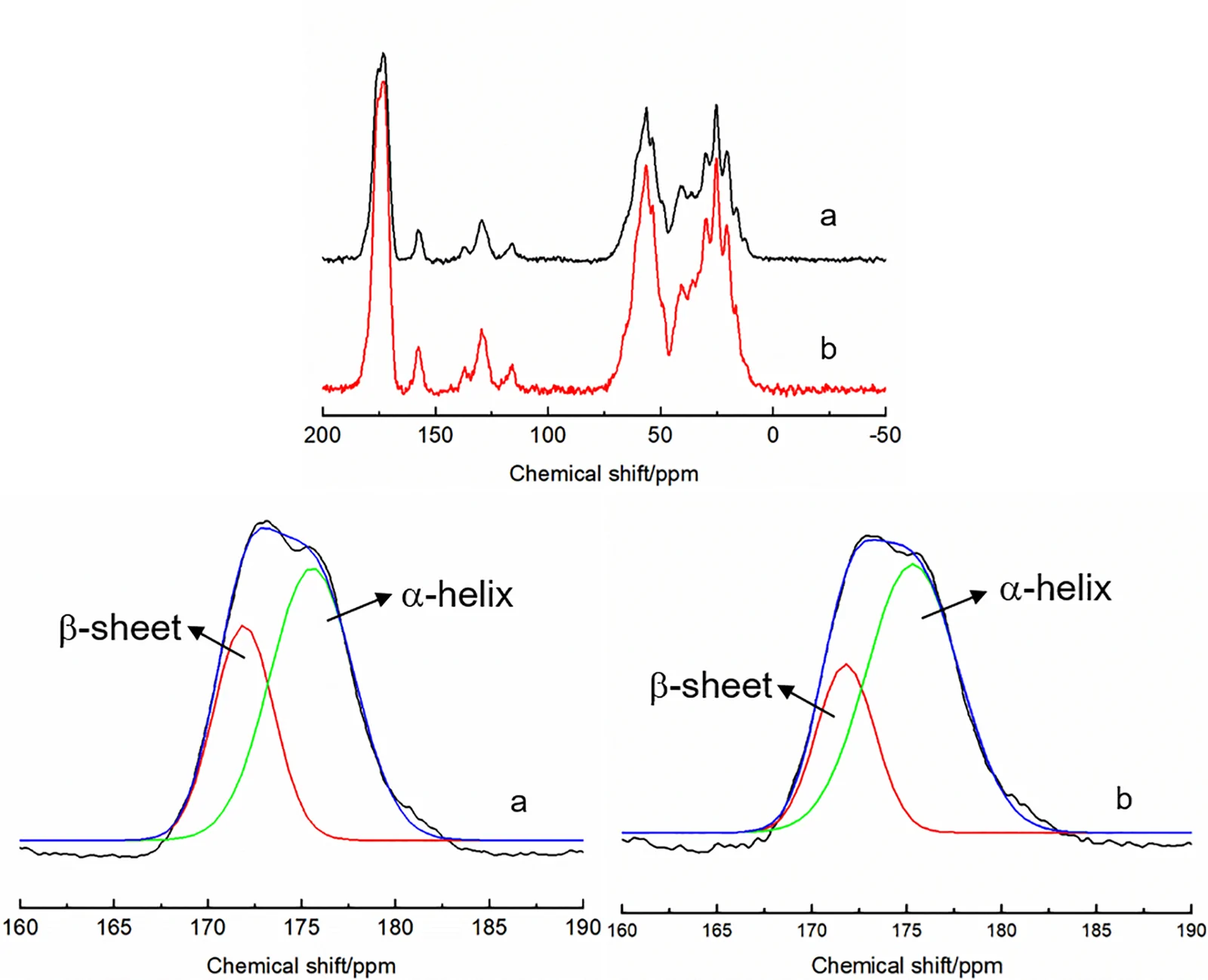
^13^C NMR of raw (a) and treated wool for (b) 5 min, and their fittings with Gaussian functions.

The high-content disulfide bonds contribute to the structural stability of keratin-associated proteins. Raman spectrum is an effective method to characterize disulfide bonds, and the area ratio of the peak at 510 cm^-1^ to the peak at 1450 cm^-1^ can be used to show the disulfide bond content [32–34]. After the calculation from Fig 5, the ratio of raw fiber is 1.26, and the ratio of wool fiber treated for 3 and 5 min decreased to 1.08 and 0.62, respectively. The results indicated that the broken disulfide bonds, which resulted in the keratin-associated proteins becoming loose and being removed.

**Fig 5:**
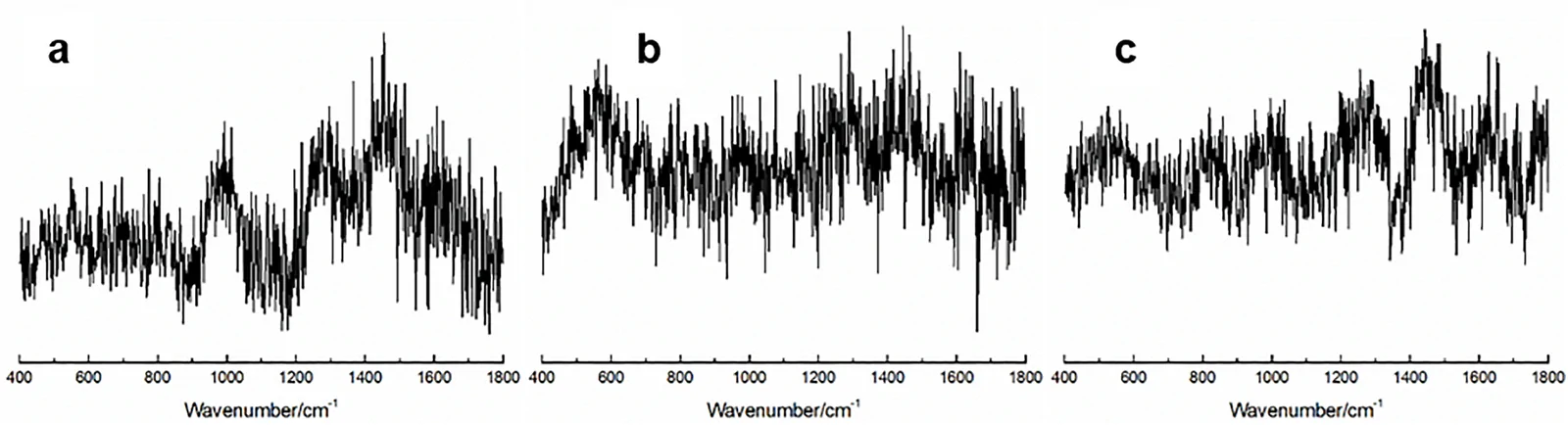
Raman of raw (a) and treated wool for (b) 3 and (c) 5 min.

### 3.3 Dissolution mechanism analysis

To study the dissolution mechanism obviously, the control and treated sample at 5 min was chosen to conduct the XPS test for the evaluation of element and bond changes. Fitting results and element compositions were listed in Fig 6 and Table 2, respectively. The C element of natural wool is about 75% due to the lipid layer at the fiber surface [35]. The element peak and composition have no difference between the raw wool and treated wool. However, compared with the raw wool, the modified wool has a lower C and S content, and a higher N and O element, indicating that the lipid layer was removed and the disulfide bond was cleaved during this process, which increased the exposure degree of keratin. The fitting results further are consistent with the above analysis. The percentage of -C-C- and –C-H- related to the lipid layer decreased with the percentage increase of –C-N- and –C = O- ascribed to the keratin. The percentage increase of –SO_x_- was due to the reduction of disulfide bonds by NADES and then the products being oxidized by the oxygen in the air [36–40]. In a word, the NADES could remove the lipid layer and break disulfide bonds, which increases the exposure degree of keratin and contributes to the increase of hydrophilic performance.

**Fig 6:**
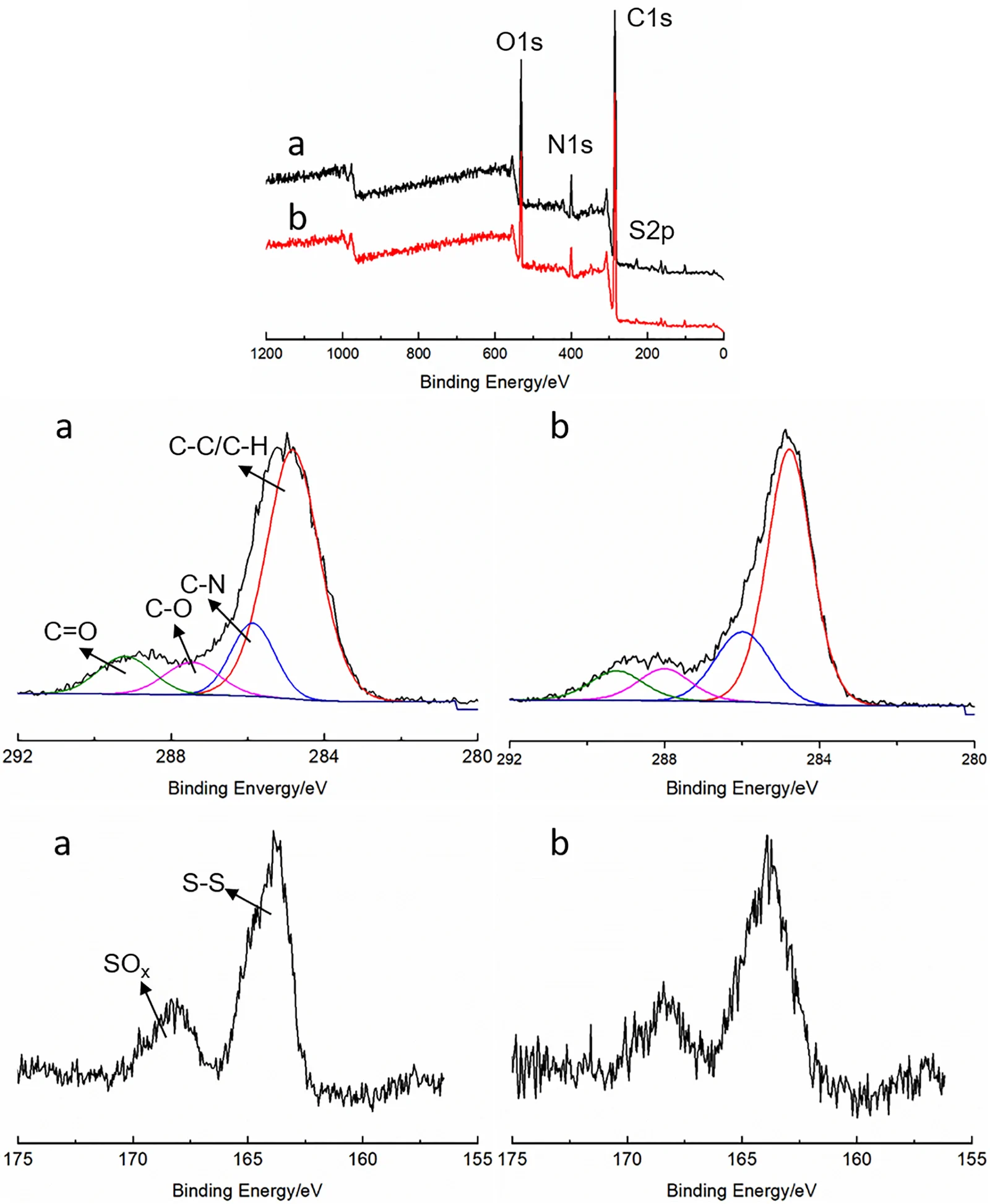
XPS spectra of raw (a) and treated wool (b) 5 min and their fittings curves.

### 3.4 Wetting performance analysis

The wetting performance test results of raw wool and modified wool were listed in Table 3. The raw wool shows hydrophobic properties with a water contact angle of 129.5^o^ and a wetting time of more than 1800s due to the lipid layer and dense scale layer. With the treatment time increase, the water contact angle and wetting time decreased. After treatment for 5 min, the water contact angle and wetting time decreased to 87.5 ^o^ and 28 s, respectively. The results indicated that NADES could modify wool surface, which increases the fiber’s hydrophilic performance.

**Table 3:** The wettability of raw wool and treated wool.

Samples	Contact angle	Wetting time
Control	129.5	>1800 s
NADES-treated (3 min)	116.9	62 s
NADES-treated (5 min)	87.5	28 s

### 3.5 Dyeing performance analysis

The dyeing performance was shown in Table 4. Due to the obstruction effect of the scale layer, the dye molecular is challenging to diffuse into the inner of the wool fiber, which results in poor dye performance at low temperature. In this study, the K/S value increased significantly from approximately 5–16, reflecting a substantial 300% increase in shade depth. According to the Kubelka-Munk theory, the K/S value is a quantitative measure of color intensity and the concentration of the colorant within the substrate; therefore, transitioning from a pale tint to a full, deep shade naturally yields a profound rise in the K/S value. This robust increase primarily indicates highly efficient dye exhaustion and deep dyeing performance. Most importantly, rigorous soap-washing data confirms that this intense coloration is attributed to genuine dye fixation rather than unfixed surface adsorption. After undergoing a standardized washing cycle, the K/S value shifted only marginally, dropping from 16.91 to 16.13. This minimal reduction mathematically corresponds to a 95.4% dye fixation rate. If the dye had remained as unbound “floating color” on the fabric surface, the K/S value would have experienced a drastic decline following the washing process. The exceptional retention of color intensity unequivocally demonstrates that the dye molecules were firmly anchored to the fiber matrix. Furthermore, the washing color fastness exhibited a minor half-grade decrease, falling from Grade 4 to Grade 3–4. It is imperative to note that a slight fastness reduction is a physically normal phenomenon when the dye concentration and shade depth are increased by 300%. When fabrics are dyed to a deep shade, the surface area is nearly fully covered with dye molecules. Under the mechanical and chemical stress of laundering, a minuscule amount of these exhaustively loaded surface dyes will inevitably detach, leading to the observed half-grade decline. Nevertheless, a Grade 3–4 fastness rating remains at an excellent practical level, ensuring the durability and reliability of the material for real-world applications.

**Table 4:** The dye ability and color fastness of raw wool and treated wool.

Samples	K/S	K/S after washing (Soap method)	Washing fastness			Rubbing fastness		Light fastness
			Staining cotton	Staining wool	Color change	Dry	Wet	
Control	1.64	1.52	4-5	5	4	4	3-4	4
NADES-treated (3 min)	5.77	5.56	4-5	5	4	4	3-4	4
NADES-treated (5 min)	16.91	16.13	4	4 ~ 5	3-4	4	3	4

### 3.6 Anti-felting performance analysis

Wool have a typical scale structure, and this produces a one-way movement in the tip-root direction, with the formation of an inextricable fiber tangle, responsible for the shrinkage of wool [41]. Due to the intact scale layer, the raw wool has a low diameter and a high density (Table 5). However, after the treatment, the wool has a higher diameter and a lower density. Furthermore, the diameter of wool treated for 5 min has surpassed the threshold value of 30 mm, which indicated that the wool meets the machine-washing standard [42]. It also shows that this method has application potential in the textile industry.

**Table 5:** The anti-felting ability of raw wool and treated wool.

Samples	Diameter (mm)	Felt-ball density (g/cm^3^)
Control	26.44	0.10
NADES-treated (3 min)	28.41	0.083
NADES-treated (5 min)	32.97	0.053

### 3.7 Method advance evaluation

The advance of the developed method was evaluated by the comparison with present methods that could make wool have satisfactory surface performance, as shown in Table 6. On the one hand, the developed method had the advantages of lower temperature and less time. On the other hand, the developed method has a shorter procedure and less usage of chemical reagents; moreover, although the enzyme had a low treatment temperature, considering the lower heat capacity and treatment time, the developed method had an advance in energy consumption [43]. Overall, the developed way was more advanced than present methods.

**Table 6:** The advance evaluation of the developed method.

Methods	Solid-liquid weight ratio	pH	Pre-treatment	Time /min	Temperature /degree Celsius
[Bmim]Cl (Ionic liquid) [44]	/	/	/	10	120
Choline chloride-urea DES (1:2 M ratio) [45]	1:4.3	/	/	122	93
Proteinase K (Enzyme^)^ [46]	1:20	8.0	H_2_O_2_, 60 min 50 ℃	120	50
This method	1:5	/	/	5	80

## 4. Conclusion

In this work, a novel, facile, and green approach for micro-dissolution treatment via NADES was proposed and then applied for the surface properties improvement of wool fiber. The results showed that the NADES could remove the lipid and scale layer on the surface of the wool, accompanied by the cleavage of the disulfide bond. Meanwhile, the secondary structure and macromolecular chain skeleton were retained during this process. Finally, the results also showed that the surface-related properties of wool, including wettability, dyeability, and felting shrinkage were improved and met the industrial standard. This work indicates that the NADES can be used for the surface performance improvement of protein materials with an application potential in large-scale production.
